# Effects of different training schedules based on distance and accelerometry measures: A full-season case study in professional soccer players

**DOI:** 10.1038/s41598-023-37337-0

**Published:** 2023-06-23

**Authors:** Hadi Nobari, Nader Alijanpour, Amirhossein Talvari, Rafael Oliveira

**Affiliations:** 1 Department of Exercise Physiology, Faculty of Educational Sciences and Psychology, University of Mohaghegh Ardabili, Ardabil, Iran; 2 Faculty of Sport Sciences, University of Extremadura, 10003 Cáceres, Spain; 3 Department of Physical Education and Sport, Faculty of Sport Science, University of Granada, 18010 Granada, Spain; 4 Department of Health and Sport Medicine, Faculty of Sports Siences, University of Tehran, 1417935840 Tehran, Iran; 5 Sports Science School of Rio Maior–Polytechnic Institute of Santarém, 2040-413 Rio Maior, Portugal; 6 Life Quality Research Centre, 2040-413 Rio Maior, Portugal; 7 Research Center in Sport Sciences, Health Sciences and Human Development, 5001-801 Vila Real, Portugal

**Keywords:** Physiology, Medical research, Engineering

## Abstract

This study aimed to evaluate external load based on distance and accelerometry measures in six different microcycle schedules with congested (CW2, CW3, and CW4) and regular weeks (RW4, RW5, and RW6) in professional soccer players. Twelve Iranian First League players (age: 28.2 ± 3.8 years; body mass: 77.92 ± 4.72 kg, and height: 183.2 ± 0.06 cm) participated in this study. A GPSPORTS system was used to collect training/match durations, total distance, distance covered at different speeds, number of accelerations, delecelerations and body load over 29 weeks, 40 matches, and 121 training sessions. Data were analysed by weekly mean and accumulated weekly values. The results showed a tendency for higher values in CWs than RWs. Regarding mean total distance, RW4 and RW5 presented lower values with very large effect size than CW2 (all, p < 0.05). The mean distance covered between 16 and 23 km/h showed that all RWs presented significantly lower values than all CWs (p < 0.05 with considerable effect size). In addition, mean acceleration values at < 2 m/s^2^ showed significantly lower values than all CWs (p < 0.05 with a very large effect size). Therefore, it is recommended to coaches pay special attention to players who participate in matches to reduce fatigue and improve the performance of professional soccer players. Ensuring recovery mechanisms are in place to minimize the external load is recommended. Coaches arrange practice sessions for non-starters who do not experience similar external loads compared to starters to prepare them for potential competition.

## Introduction

Soccer is a popular team sport and a very challenging activity^1^. Thus soccer players cover a distance of about 10 to 15 km per match, from which around 1 km is performed with high intensity^2^ while in training, they covered a total distance between 2.143–9.540 km and only a range 0.410–1.884 km is covered in running (i.e. > 14 km/h), 7–541 m in high-speed running (> 18 km/h), and 1–190 m in sprinting (i.e. > 24 km/h)^3^.

Professional soccer teams must compete in as many as 50 matches per season^4^. In addition, the elite teams generally compete for domestic leagues and country-wide championships, resulting in periods of congested fixtures^5^. Accumulated competition in a short period potentially leads to residual fatigue and poor performance due to insufficient physical and cognitive recovery time^6^. Matches have a lasting fatigue effect on players, lasting up to 72 h^7^. As a result, a greater understanding of how players who play numerous weekly matches deal with external expectations is required^5^.

Numerous studies have examined the effects of congested fixtures on soccer players, but there is still a discrepancy regarding the dependence of some GPS metrics regarding congested schedules that need to be resolved^8^. The training load can control personalization of the training process measures^9^, which can be examined in two parts: (i) the external load and (ii) the internal load, which is related to psychobiological reactions to the external load^10,11^.

Quantifying and evaluating the training load experienced during microcycles with a different number of matches could help explain the changes among microcycles throughout the competitive season. Indeed, a high variety of matches and day-by-day training sessions can lead to increased levels of fatigue and a higher risk of illness and injury^12^. Therefore, monitoring the training load to ensure optimal match-day performance and recovery is crucial^13,14^. More matches per week, and consequently a higher number of matches per season, can make it hard for coaches to manage the training load and avoid accumulated fatigue while ensuring that players remain at optimal physical fitness. The improper training load management has quickly become a primary risk factor in no-contact injuries^15^. Usually, management load is considered in weekly microcycles consisting of one match per week, though it is noteworthy that elite soccer players often play two or three matches in seven days^16^.

Research has shown that competition is the most challenging microcycle session^17^. The microcycles are weekly training periods, which depend from the day after the match to the subsequent match, whose duration can also vary depending on the competitive calendar^18,19^. The training load is gradually reduced from the four days left before the match to the day, which means that the load on the first day is very different from the fourth day before match^20^. Specifically, previous research confirmed that the duration of the microcycle might also affect a load of soccer players^6,12,18,21–24^ but only two studies used mean or accumulated external load values and explained the number of the matches in the microcycle load values^12,21^ while the other used different metrics or did not clarify the number of matches^6,18,22–24^. Furthermore, none of the mentioned studies used both mean and accumulated data values which could provide clear insights for coaches and their staff.

Specifically, it has been shown that the total accumulated distance (including match and training load) was higher in a week with two matches compared to a week with one match. On the other hand, the daily training total distance was lower in the three-match week versus the one- and two-match weeks, though the accumulative weekly distance was highest in this week^21^. Moreover, distance of 19–23.9 km/h showed higher values in the three-match week than one match week, while distance > 24 km/h showed no differences regardless of the match number per week^12^. Nonetheless, these studies only used three to five microcycles and lacked using accelerometry-based variables^12,21^.

A correct understanding of the external load information of training and competition in professional football players allows coaches to properly adjust training sessions during the training microcycle with different numbers of matches. It is important to examine further how the training load is managed on weeks with one, two, and three matches where the weekly training frequency could be five, four, and two, respectively)^12^.

Therefore, the purpose of this study was to compare distance and accelerometry-based measures in six different microcycle schedules with both regular and congested weeks in professional soccer players, using daily mean and weekly accumulated data values. The research hypothesis is that the higher accumulated and mean external load may occurr in the congested weeks^12,21^.

## Material and methods

### Experimental approach to the problem

This is a descriptive-longitudinal study. During the season of 2019/20, professional soccer players were monitored for 43 weeks through a GPS. For this study, weeks were classified as either regular (with one match per week, RW) or congested (with two matches per week, CW). Fourteen weeks were not included in the analysis due to the inclusion criteria of participants not being followed or due to a lack of official matches in those weeks. Table 1 describes all microcycles schedules included in the study.

**Table 1:** Number of sessions and matches of each microcycle schedule.

	RW4	RW5	RW6	CW2	CW3	CW4
Weeks (n)	11	5	4	3	2	4
Total sessions (n)	44	25	24	6	6	16
Total matches (n)	11	5	4	6	4	8
Playing time (min)	65	73	73	83	81	68
Participation in matches (n)	8	4	3	2	2	3

*RW4* regular weeks with four training sessions, *RW5* regular weeks with five training sessions, *RW6* regular weeks with six training sessions, *CW2* congested weeks with two training sessions, *CW3* congested weeks with three training sessions, *CW4* congested weeks with four training sessions.

### Participants

Following previous studies with small sample sizes^12,21^, twelve professional male players (age: 28.2 ± 3.8 years; body mass: 77.9 ± 4.7 kg; height: 183.2 ± 0.1 cm; experience as professionals: at least 5 years of training in professional teams) from Iranian First League participated in this study. The position of participating players were central defenders (n = 3), wide defenders (n = 3), midfielders (n = 2), wide midfielders (n = 2), and forwards (n = 2). To be included in the study, the following inclusion criteria were defined: minimum of one full match in each type of the six microcycles schedules included and at least 90% of the training sessions during each microcycle. Players who missed two consecutive weeks of training due to injury or illness were excluded from the study.

Players received detailed information about the study design, procedures, and methodological approach and signed free participation consent. Ethical standards of the Declaration of Helsinki were followed while research ethics committee approved the study. Before beginning the study, the research ethics committee at Ardabil University of Medical Sciences presented approval. All players were briefed about the study’s intent and signed an informed consent form to participate.

### External load monitoring

The GPSPORTS system (Pty Ltd, Model: SPI High-Performance Unit, Australian) was used to collect all data. This device includes the following characteristics: 15 Hz for location, 100 Hz for accelerometry, and 50 Hz for the magnetometer. It consists of the following dimensions: 74 mm × 42 mm × 16 mm and 56 g of weight. It is also waterproof. This device has presented high validity and reliability (98%) in a previous study^25^.

To collect data, the following procedures were applied to collect data: (1) before the start of each session (training or match), belts were placed on the players’ shoulders and chest (all players used the same belt for all season); (2) after each session, all belts were collected; (3) then, belts were put in the dock system to download the data; (4) data was saved on a laptop using the Team AMS R1 2019.1 software, developed by GPSports, based in Canberra, Australia; (5) after the previous procedures, all data was erased from the belt; (6) before next session, all devices were recharged. Our study followed the manufacturer's guidelines and protocols for calibrating the device before the data collection session. This involved checking and adjusting the distance, speed, and acceleration parameters to align with known standards or reference measurements.

For data analysis, the following variables were used: total training and match duration, total distance, distance covered between 0 and 5.9 km/h (D1), distance covered between 6 and 9.9 km/h (D2), distance covered between 10 and 15.9 km/h (D3), distance covered between 16 and 23 km/h (D4), distance covered > 23 km/h (D5) Accelerations Zone1 (< 2 m/s^2^) (AccZ1); Accelerations Zone2 (2–4 m/s^2^) (AccZ2); Accelerations Zone3 (> 4 m/h^2^) (AccZ3); Decelerations Zone1 (< − 2 m/h^2^) (DecZ1); Decelerations Zone2 (− 2 to − 4 m/h^2^) (DecZ2); Decelerations Zone3 (> − 4 m/h^2^) (DecZ3) and body load. We opted to use “distance” instead of other walking or running definition based on a recent systematic review^3^. Accelerometery-based thresholds were defined according to previous studies^27,28^. Each microcycle schedule presented all variables by daily mean (average of all sessions of the microcycle) and accumulated mean weekly values (average of the sum of each microcycle session).

### Statistical analysis

Data were analysed using SPSS version 23.0 (SPSS Inc., Chicago, IL) for Windows statistical software package. The cutoff esteem was set to be p ≤ 0.05 for all measures to represent statistical significance. Initially, descriptive statistics were used to describe and characterize the sample through means ± standard deviation (SD). Shapiro–Wilk test was used to analyse assumption of normality. The variables of weekly D1, weekly AccZ2, weekly DecZ3, weekly BL, accumulated AccZ1, accumulated DecZ2, accumulated DecZ3, accumulated BL did not present normal distribution and consequently were analysed with ANOVA Friedman and Wilcoxon tests. The remaining variables were analysed through repeated measures ANOVA with Bonferroni post hoc to compare each microcycle schedule. The Hedge effect-size (ES) was calculated to determine the magnitude of effects by standardizing the coefficients according to the appropriate between-subjects standard deviation and was assessed using the following criteria: < 0.2 = trivial, 0.2 to 0.6 = small effect, 0.6 to 1.2 = moderate effect, 1.2 to 2.0 = large effect and > 2.0 = very large effect^29^.

### Ethics approval and consent to participants

The players and their staff coach signed an informed consent form to engage in this study. The study has been approved by the Ardabil University of medical sciences Ethics Committee before its start (R.ARUMS.REC.1399.546), and the Helsinki Declaration was used to follow the recommendations of Human Ethics in Research.

## Results

Both parametric and non-parametric ANOVAs presented significant differences among all microcycles for weekly mean values (p < 0.05 for all measures). Specifically, the following main effects were found: duration [F(5) = 20.642, p < 0.001, η^2^_*p*_ = 0.652]; total distance [F(5) = 15.057, p < 0.001, η^2^_*p*_ = 0.578]; D1 [t(5) = 46.381, p < 0.001]; D2 [F(5) = 11.391, p < 0.001, η^2^_*p*_ = 0.509]; D3 [F(5) = 20.688, p < 0.001, η^2^_*p*_ = 0.653]; D4 [F(5) = 16.917, p < 0.001, η^2^_*p*_ = 0.606]; D5 [F(5) = 7.575, p < 0.001, η^2^_*p*_ = 0.408]; AccZ1 [F(5) = 12.680, p < 0.001, η^2^_*p*_ = 0.535]; AccZ2 [t(5) = 23.789, p < 0.001]; AccZ3 [F(5) = 3.936, p = 0.004, η^2^_*p*_ = 0.264]; DecZ1 [F(5) = 9.062, p < 0.001, η^2^_*p*_ = 0.452]; DecZ2 [F(5) = 7.632, p < 0.001, η^2^_*p*_ = 0.410]; DecZ3 [t(5) = 25.000, p < 0.001]; BL [t(5) = 35.000, p < 0.001].

Table 2 presents external data comparisons based on weekly mean values between all microcycles. Specifically, the following main effects were found: duration [F(5) = 8.742, p < 0.001, η^2^_*p*_ = 0.443]; total distance [F(5) = 16.689, p < 0.001, η^2^_*p*_ = 0.603]; D1 [F(5) = 27.630, p < 0.001, η^2^_*p*_ = 0.715]; D2 [F(5) = 6.653, p < 0.001, η^2^_*p*_ = 0.377]; D3 [F(5) = 5.045, p < 0.001, η^2^_*p*_ = 0.314]; D4 [F(5) = 1.174, p = 0.334, η^2^_*p*_ = 0.096]; D5 [F(5) = 10.114, p < 0.001, η^2^_*p*_ = 0.479]; AccZ1 [t(5) = 20.143, p < 0.001]; AccZ2 [F(5) = 11.161, p < 0.001, η^2^_*p*_ = 0.504]; AccZ3 [F(5) = 14.717, p < 0.001, η^2^_*p*_ = 0.572]; DecZ1 [F(5) = 13.205, p < 0.001, η^2^_*p*_ = 0.546; DecZ2 [t(5) = 32.905, p < 0.001]; DecZ3 [t(5) = 27.530, p < 0.001]; BL [F(5) = 3.247, p < 0.012, η^2^_*p*_ = 0.228].

**Table 2:** Comparison of six microcycles schedules by weekly mean values.

Variables	RW4	RW5	RW6	CW2	CW3	CW4	p-value	Effect size
Duration (min)	72 ± 1	73 ± 1	71 ± 1	86 ± 3	109 ± 8	80 ± 2	RW4 vs. RW5: > 0.999 RW4 vs. RW6: > 0.999 RW4 vs. CW2: 0.004 RW4 vs. CW3: 0.008 RW4 vs. CW4: 0.074 RW5 vs. RW6: > 0.999 RW5 vs. CW2: 0.012 RW5 vs. CW3: 0.005 RW5 vs. CW4: 0.071 RW6 vs. CW2: 0.004 RW6 vs. CW3: 0.005 RW6 vs. CW4: 0.017 CW2 vs. CW3: 0.061 CW2 vs. CW4: 0.878 CW3 vs. CW4: 0.027	– – − 6.04 − 6.27 – – − 5.61 − 6.10 – − 6.48 − 6.44 − 5.50 – – 4.80
Total distance (m)	6933 ± 218	6169 ± 181	5358 ± 117	9468 ± 536	7011 ± 587	6881 ± 254	RW4 vs. RW5: 0.398 RW4 vs. RW6: < 0.001 RW4 vs. CW2: 0.015 RW4 vs. CW3: > 0.999 RW4 vs. CW4: > 0.999 RW5 vs. RW6: 0.024 RW5 vs. CW2: 0.001 RW5 vs. CW3: > 0.999 RW5 vs. CW4: 0.295 RW6 vs. CW2: < 0.001 RW6 vs. CW3: 0.247 RW6 vs. CW4: 0.001 CW2 vs. CW3: 0.314 CW2 vs. CW4: 0.006 CW3 vs. CW4: > 0.999	– 8.69 − 5.98 – – 5.14 − 7.96 – – − 10.23 – − 7.44 – 5.96 –
D1 (m)	1602 ± 33	1693 ± 54	3432 ± 16	1675 ± 47	2103 ± 121	3170 ± 180	RW4 vs. RW5: 0.218 RW4 vs. RW6: < 0.001 RW4 vs. CW2: > 0.999 RW4 vs. CW3: 0.020 RW4 vs. CW4: < 0.001 RW5 vs. RW6: < 0.001 RW5 vs. CW2: > 0.999 RW5 vs. CW3: 0.149 RW5 vs. CW4: < 0.001 RW6 vs. CW2: < 0.001 RW6 vs. CW3: < 0.001 RW6 vs. CW4: > 0.999 CW2 vs. CW3: 0.112 CW2 vs. CW4: < 0.001 CW3 vs. CW4: 0.002	– − 20.72 – − 5.45 − 11.70 − 18.56 – – − 10.73 19.17 10.83 – – − 10.97 − 6.72
D2 (m)	1377 ± 42	1412 ± 53	1371 ± 54	1542 ± 96	2170 ± 205	1639 ± 83	RW4 vs. RW5: > 0.999 RW4 vs. RW6: > 0.999 RW4 vs. CW2: > 0.999 RW4 vs. CW3: 0.039 RW4 vs. CW4: 0.009 RW5 vs. RW6: > 0.999 RW5 vs. CW2: > 0.999 RW5 vs. CW3: 0.033 RW5 vs. CW4: 0.169 RW6 vs. CW2: 0.957 RW6 vs. CW3: 0.018 RW6 vs. CW4: 0.009 CW2 vs. CW3: 0.153 CW2 vs. CW4: > 0.999 CW3 vs. CW4: 0.469	– – – − 5.17 − 3.85 – – − 4.89 – – − 5.15 − 3.70 – – –
D3 (m)	1000 ± 26	937 ± 54	1202 ± 57	1677 ± 144	2279 ± 242	1274 ± 71	RW4 vs. RW5: > 0.999 RW4 vs. RW6: 0.004 RW4 vs. CW2: 0.007 RW4 vs. CW3: 0.004 RW4 vs. CW4: 0.009 RW5 vs. RW6: 0.033 RW5 vs. CW2: 0.004 RW5 vs. CW3: 0.001 RW5 vs. CW4: 0.040 RW6 vs. CW2: 0.042 RW6 vs. CW3:0.013 RW6 vs. CW4: 0.765 CW2 vs. CW3: 0.555 CW2 vs. CW4: 0.086 CW3 vs. CW4: 0.022	– − 4.40 − 6.32 − 7.18 − 4.95 − 4.61 − 6.57 − 7.39 − 5.16 − 4.19 − 5.91 – – – 5.44
D4 (m)	452 ± 26	429 ± 28	431 ± 30	743 ± 62	856 ± 91	567 ± 46	RW4 vs. RW5: > 0.999 RW4 vs. RW6: > 0.999 RW4 vs. CW2: 0.001 RW4 vs. CW3: 0.028 RW4 vs. CW4: 0.026 RW5 vs. RW6: > 0.999 RW5 vs. CW2: < 0.001 RW5 vs. CW3: 0.009 RW5 vs. CW4: 0.028 RW6 vs. CW2: < 0.001 RW6 vs. CW3: 0.017 RW6 vs. CW4: 0.004 CW2 vs. CW3: > 0.999 CW2 vs. CW4: 0.011 CW3 vs. CW4: 0.245	– – − 5.91 − 5.83 − 2.97 – − 6.30 − 6.12 − 3.50 − 6.19 − 6.06 − 3.38 – 3.11 –
D5 (m)	121 ± 16	156 ± 12	77 ± 10	154 ± 20	181 ± 24	163 ± 20	RW4 vs. RW5: 0.434 RW4 vs. RW6: 0.010 RW4 vs. CW2: 0.279 RW4 vs. CW3: 0.728 RW4 vs. CW4: 0.009 RW5 vs. RW6: 0.001 RW5 vs. CW2: > 0.999 RW5 vs. CW3: > 0.999 RW5 vs. CW4: > 0.999 RW6 vs. CW2: 0.001 RW6 vs. CW3: 0.017 RW6 vs. CW4: 0.001 CW2 vs. CW3: > 0.999 CW2 vs. CW4: > 0.999 CW3 vs. CW4: > 0.999	– 3.18 – – − 2.24 6.91 – – – − 4.70 − 5.46 − 5.25 – – –
AccZ1 (nr)	72 ± 3	71 ± 3	68 ± 2	81 ± 5	115 ± 10	91 ± 5	RW4 vs. RW5: > 0.999 RW4 vs. RW6: > 0.999 RW4 vs. CW2: 0.881 RW4 vs. CW3: 0.048 RW4 vs. CW4: 0.002 RW5 vs. RW6: > 0.999 RW5 vs. CW2: 0.842 RW5 vs. CW3: 0.024 RW5 vs. CW4: 0.008 RW6 vs. CW2: 0.122 RW6 vs. CW3: 0.015 RW6 vs. CW4: 0.002 CW2 vs. CW3: 0.178 CW2 vs. CW4: 0.271 CW3 vs. CW4: 0.914	– – – − 5.62 − 4.45 – – − 5.75 − 4.68 – − 6.29 − 5.83 – –
AccZ2 (nr)	25 ± 1	24 ± 2	21 ± 1	23 ± 2	32 ± 3	28 ± 2	RW4 vs. RW5: > 0.999 RW4 vs. RW6: 0.041 RW4 vs. CW2: > 0.999 RW4 vs. CW3: > 0.999 RW4 vs. CW4: 0.076 RW5 vs. RW6: 0.411 RW5 vs. CW2: > 0.999 RW5 vs. CW3: > 0.999 RW5 vs. CW4: 0.140 RW6 vs. CW2: > 0.999 RW6 vs. CW3: 0.171 RW6 vs. CW4: 0.001 CW2 vs. CW3: 0.478 CW2 vs. CW4: 0.120 CW3 vs. CW4: > 0.999	– 3.86 – – – – – – – – – − 4.27 – – –
AccZ3 (nr)	3 ± 0.4	4 ± 1	3 ± 0.4	3 ± 0.4	3 ± 0.3	4 ± 1	RW4 vs. RW5: 0.129 RW4 vs. RW6: > 0.999 RW4 vs. CW2: > 0.999 RW4 vs. CW3: > 0.999 RW4 vs. CW4: 0.207 RW5 vs. RW6: 0.177 RW5 vs. CW2: 0.033 RW5 vs. CW3: 0.701 RW5 vs. CW4: > 0.999 RW6 vs. CW2: > 0.999 RW6 vs. CW3: > 0.999 RW6 vs. CW4: 0.696 CW2 vs. CW3: > 0.999 CW2 vs. CW4: 0.216 CW3 vs. CW4: > 0.999	– – – – – – 1.27 – – – – – – – –
DecZ1 (nr)	44 ± 2	48 ± 3	36 ± 1	42 ± 3	59 ± 5	58 ± 4	RW4 vs. RW5: 0.367 RW4 vs. RW6: 0.031 RW4 vs. CW2: > 0.999 RW4 vs. CW3: 0.777 RW4 vs. CW4: 0.003 RW5 vs. RW6: 0.008 RW5 vs. CW2: 0.694 RW5 vs. CW3: > 0.999 RW5 vs. CW4: 0.132 RW6 vs. CW2: 0.135 RW6 vs. CW3: 0.015 RW6 vs. CW4: 0.001 CW2 vs. CW3: 0.315 CW2 vs. CW4: 0.008 CW3 vs. CW4: > 0.999	– 4.89 – – − 4.27 5.18 – – – – − 6.16 − 7.29 – − 4.37 –
DecZ2 (nr)	16 ± 1	17 ± 2	11 ± 1	16 ± 1	21 ± 2	19 ± 1	RW4 vs. RW5: 0.733 RW4 vs. RW6: 0.031 RW4 vs. CW2: > 0.999 RW4 vs. CW3: 0.796 RW4 vs. CW4: 0.016 RW5 vs. RW6: 0.016 RW5 vs. CW2: > 0.999 RW5 vs. CW3: > 0.999 RW5 vs. CW4: > 0.999 RW6 vs. CW2: 0.017 RW6 vs. CW3: 0.004 RW6 vs. CW4: < 0.001 CW2 vs. CW3: 0.438 CW2 vs. CW4: 0.580 CW3 vs. CW4: > 0.999	– 4.83 – – − 2.90 3.66 – – – − 4.83 − 6.11 − 7.72 – – –
DecZ3 (nr)	5 ± 1	6 ± 1	3 ± 1	4 ± 1	5 ± 1	6 ± 0.4	RW4 vs. RW5: 0.758 RW4 vs. RW6: 0.105 RW4 vs. CW2: > 0.999 RW4 vs. CW3: > 0.999 RW4 vs. CW4: 0.100 RW5 vs. RW6: 0.013 RW5 vs. CW2: > 0.999 RW5 vs. CW3: > 0.999 RW5 vs. CW4: > 0.999 RW6 vs. CW2: > 0.999 RW6 vs. CW3: 0.030 RW6 vs. CW4: 0.003 CW2 vs. CW3: > 0.999 CW2 vs. CW4: 0.227 CW3 vs. CW4: > 0.999	– – – – – 2.90 – – – – − 1.93 − 3.80 – – –
BL (AU)	137 ± 18	123 ± 9	170 ± 31	188 ± 22	261 ± 28	162 ± 9	RW4 vs. RW5: > 0.999 RW4 vs. RW6: > 0.999 RW4 vs. CW2: 0.226 RW4 vs. CW3: 0.011 RW4 vs. CW4: > 0.999 RW5 vs. RW6: > 0.999 RW5 vs. CW2: 0.043 RW5 vs. CW3: 0.004 RW5 vs. CW4: 0.026 RW6 vs. CW2: > 0.999 RW6 vs. CW3: 0.355 RW6 vs. CW4: > 0.999 CW2 vs. CW3: 0.314 CW2 vs. CW4: > 0.999 CW3 vs. CW4: 0.058	– – – − 5.09 – – − 3.73 − 6.41 − 4.18 – – – – – –

*RW4* regular weeks with four training sessions, *RW5* regular weeks with five training sessions, *RW6* regular weeks with six training sessions, *CW2* congested weeks with two training sessions, *CW3* congested weeks with three training sessions, *CW4* congested weeks with four training sessions, *D1* distance covered between 0 and 5.9 km/h, *D2* distance covered between 6 and 9.9 km/h, *D3* distance covered between 10 and 15.9 km/h, *D4* distance covered between 16 and 23 km/h, *D5* distance covered between > 23 km/h, *AccZ1* Accelerations Zone1 (< 2 m/s^2^), *AccZ2* Accelerations Zone2 (2 to 4 m/s^2^), *AccZ3* Accelerations Zone3 (> 4 m/h^2^), *DecZ1* Decelerations Zone1 (< − 2 m/h^2^), *DecZ2* Decelerations Zone2 (− 2 to − 4 m/h^2^), *DecZ3* Decelerations Zone3 (> − 4 m/h^2^), *BL* body load.

To simplify the description of pairwise comparisons in both tables, only Hedge ES for significant p-values were reported. In this regard, all ES mean very large effect.

In addition, both parametric and non-parametric ANOVA’s present significant differences among all microcycles for accumulated mean values (p < 0.05) for all measures except for D4 and BL (both, p > 0.05). Table 3 presents external data comparisons between all microcycles based on weekly mean values (accumulated mean of all weeks included for each microcycle). To simplify the description, only effect sizes for significant p-values were reported and all mean enormous effects.

**Table 3:** Comparison of the six microcycles schedules by accumulated mean values.

Variables	RW4	RW5	RW6	CW2	CW3	CW4	p-value	Effect size
Duration (min)	282 ± 11	330 ± 16	337 ± 19	247 ± 19	245 ± 10	317 ± 17	RW4 vs. RW5: 0.043 RW4 vs. RW6: 0.203 RW4 vs. CW2: > 0.999 RW4 vs. CW3: 0.694 RW4 vs. CW4: 0.095 RW5 vs. RW6: > 0.999 RW5 vs. CW2: 0.080 RW5 vs. CW3: 0.038 RW5 vs. CW4: > 0.999 RW6 vs. CW2: 0.080 RW6 vs. CW3: 0.038 RW6 vs. CW4: > 0.999 CW2 vs. CW3: > 0.999 CW2 vs. CW4: 0.156 CW3 vs. CW4: 0.114	− 3.73 – – – – – – 6.15 – – 5.85 – – – –
Total distance (m)	12,262 ± 769	14,944 ± 1265	25,052 ± 1402	19,585 ± 1570	20,182 ± 1010	22,953 ± 1408	RW4 vs. RW5: 0.068 RW4 vs. RW6: < 0.001 RW4 vs. CW2: 0.019 RW4 vs. CW3: 0.005 RW4 vs. CW4: < 0.001 RW5 vs. RW6: 0.001 RW5 vs. CW2: 0.949 RW5 vs. CW3: 0.358 RW5 vs. CW4: 0.003 RW6 vs. CW2: 0.316 RW6 vs. CW3: 0.434 RW6 vs. CW4: > 0.999 CW2 vs. CW3: > 0.999 CW2 vs. CW4: > 0.999 CW3 vs. CW4: > 0.999	– − 10.95 − 5.73 − 8.56 − 9.13 − 7.31 – – − 5.78 – – – – – –
D1 (m)	2341 ± 116	4421 ± 404	4261 ± 296	7351 ± 282	9183 ± 675	6078 ± 699	RW4 vs. RW5: 0.003 RW4 vs. RW6: 0.003 RW4 vs. CW2: < 0.001 RW4 vs. CW3: < 0.001 RW4 vs. CW4: 0.003 RW5 vs. RW6: > 0.999 RW5 vs. CW2: < 0.001 RW5 vs. CW3: 0.001 RW5 vs. CW4: > 0.999 RW6 vs. CW2: < 0.001 RW6 vs. CW3: < 0.001 RW6 vs. CW4: 0.496 CW2 vs. CW3: 0.248 CW2 vs. CW4: > 0.999 CW3 vs. CW4: 0.290	− 6.76 − 8.95 − 22.43 − 13.64 − 7.20 – − 8.12 − 8.27 – − 10.32 − 9.12 – – – –
D2 (m)	5335 ± 270	5996 ± 373	6472 ± 452	4360 ± 372	4818 ± 281	6152 ± 456	RW4 vs. RW5: 0.930 RW4 vs. RW6: 0.276 RW4 vs. CW2: 0.657 RW4 vs. CW3: > 0.999 RW4 vs. CW4: 0.365 RW5 vs. RW6: > 0.999 RW5 vs. CW2: 0.221 RW5 vs. CW3:0.449 RW5 vs. CW4: > 0.999 RW6 vs. CW2: 0.018 RW6 vs. CW3:0.117 RW6 vs. CW4: > 0.999 CW2 vs. CW3: > 0.999 CW2 vs. CW4: 0.036 CW3 vs. CW4: 0.520	– – – – – – – – – 4.93 – – – − 4.16 –
D3 (m)	3810 ± 137	3931 ± 244	5613 ± 302	4670 ± 476	5027 ± 358	4729 ± 288	RW4 vs. RW5: > 0.999 RW4 vs. RW6: 0.002 RW4 vs. CW2: > 0.999 RW4 vs. CW3: 0.304 RW4 vs. CW4: 0.061 RW5 vs. RW6: 0.048 RW5 vs. CW2: > 0.999 RW5 vs. CW3: 0.252 RW5 vs. CW4: > 0.999 RW6 vs. CW2: > 0.999 RW6 vs. CW3: > 0.999 RW6 vs. CW4: 0.312 CW2 vs. CW3: > 0.999 CW2 vs. CW4: > 0.999 CW3 vs. CW4: > 0.999	– − 7.42 – – – − 5.92 – – – – – – – – –
D4 (m)	1712 ± 124	1798 ± 137	2027 ± 174	2010 ± 227	1912 ± 174	2099 ± 188	RW4 vs. RW5: > 0.999 RW4 vs. RW6: 0.816 RW4 vs. CW2: > 0.999 RW4 vs. CW3: > 0.999 RW4 vs. CW4: 0.075 RW5 vs. RW6: > 0.999 RW5 vs. CW2: > 0.999 RW5 vs. CW3: > 0.999 RW5 vs. CW4: > 0.999 RW6 vs. CW2: > 0.999 RW6 vs. CW3: > 0.999 RW6 vs. CW4: > 0.999 CW2 vs. CW3: > 0.999 CW2 vs. CW4: > 0.999 CW3 vs. CW4: > 0.999	– – – – – – – – – – – – – – –
D5 (m)	429 ± 64	595 ± 52	316 ± 48	381 ± 64	391 ± 54	592 ± 81	RW4 vs. RW5: 0.046 RW4 vs. RW6: 0.138 RW4 vs. CW2: > 0.999 RW4 vs. CW3: > 0.999 RW4 vs. CW4: 0.003 RW5 vs. RW6: 0.001 RW5 vs. CW2: 0.022 RW5 vs. CW3: 0.186 RW5 vs. CW4: > 0.999 RW6 vs. CW2: > 0.999 RW6 vs. CW3: > 0.999 RW6 vs. CW4: 0.006 CW2 vs. CW3: > 0.999 CW2 vs. CW4: 0.006 CW3 vs. CW4: 0.267	− 2.75 – – – − 2.16 5.38 3.54 – – – – − 4.00 – − 2.79 –
AccZ1 (nr)	282 ± 17	301 ± 18	308 ± 17	221 ± 20	256 ± 12	338 ± 26	RW4 vs. RW5: > 0.999 RW4 vs. RW6: > 0.999 RW4 vs. CW2: 0.452 RW4 vs. CW3: > 0.999 RW4 vs. CW4: 0.131 RW5 vs. RW6: > 0.999 RW5 vs. CW2: 0.196 RW5 vs. CW3: 0.724 RW5 vs. CW4: > 0.999 RW6 vs. CW2: 0.046 RW6 vs. CW3: 0.864 RW6 vs. CW4: > 0.999 CW2 vs. CW3: > 0.999 CW2 vs. CW4: 0.003 CW3 vs. CW4:0.155	– – – – – – – – – 4.53 – – – − 4.87 –
AccZ2 (nr)	94 ± 5	102 ± 9	100 ± 9	61 ± 5	72 ± 5	106 ± 9	RW4 vs. RW5: > 0.999 RW4 vs. RW6: > 0.999 RW4 vs. CW2: 0.002 RW4 vs. CW3: 0.152 RW4 vs. CW4: 0.736 RW5 vs. RW6: > 0.999 RW5 vs. CW2: 0.023 RW5 vs. CW3: 0.135 RW5 vs. CW4: > 0.999 RW6 vs. CW2: 0.013 RW6 vs. CW3: 0.133 RW6 vs. CW4: > 0.999 CW2 vs. CW3: > 0.999 CW2 vs. CW4: 0.001 CW3 vs. CW4: 0.046	– – 6.37 – – – 5.44 – – 5.17 – – – − 5.97 − 3.65
AccZ3 (nr)	13 ± 2	18 ± 2	13 ± 2	7 ± 1	6 ± 1	15 ± 2	RW4 vs. RW5: 0.020 RW4 vs. RW6: > 0.999 RW4 vs. CW2: 0.027 RW4 vs. CW3: 0.075 RW4 vs. CW4: > 0.999 RW5 vs. RW6: 0.307 RW5 vs. CW2: 0.001 RW5 vs. CW3: 0.009 RW5 vs. CW4: 0.145 RW6 vs. CW2: 0.053 RW6 vs. CW3: 0.064 RW6 vs. CW4: > 0.999 CW2 vs. CW3: > 0.999 CW2 vs. CW4: 0.003 CW3 vs. CW4: 0.036	− 2.41 – 3.66 – – – 6.72 7.33 – – – – – − 4.89 − 5.50
DecZ1 (nr)	175 ± 14	203 ± 15	166 ± 12	110 ± 9	129 ± 7	217 ± 19	RW4 vs. RW5: 0.105 RW4 vs. RW6: > 0.999 RW4 vs. CW2: 0.013 RW4 vs. CW3: 0.425 RW4 vs. CW4: 0.103 RW5 vs. RW6: 0.683 RW5 vs. CW2: 0.003 RW5 vs. CW3: 0.027 RW5 vs. CW4: > 0.999 RW6 vs. CW2: 0.024 RW6 vs. CW3: 0.535 RW6 vs. CW4: 0.652 CW2 vs. CW3: > 0.999 CW2 vs. CW4: 0.001 CW3 vs. CW4: 0.023	– – 5.33 – – – 7.26 6.10 – 5.10 – – – − 11.48 − 10.54
DecZ2 (nr)	59 ± 6	73 ± 7	51 ± 4	35 ± 3	42 ± 2	71 ± 6	RW4 vs. RW5: 0.027 RW4 vs. RW6: > 0.999 RW4 vs. CW2: 0.017 RW4 vs. CW3: 0.398 RW4 vs. CW4: 0.030 RW5 vs. RW6: 0.064 RW5 vs. CW2: 0.005 RW5 vs. CW3: 0.032 RW5 vs. CW4: > 0.999 RW6 vs. CW2: 0.015 RW6 vs. CW3: > 0.999 RW6 vs. CW4: 0.086 CW2 vs. CW3: 0.788 CW2 vs. CW4: < 0.001 CW3 vs. CW4: 0.015	− 2.07 – 4.89 – − 1.93 – 6.81 5.81 – 4.37 – – – − 7.33 − 6.26
DecZ3 (nr)	20 ± 2	25 ± 3	15 ± 2	12 ± 2	12 ± 1	23 ± 2	RW4 vs. RW5: 0.051 RW4 vs. RW6: 0.383 RW4 vs. CW2: 0.248 RW4 vs. CW3: 0.158 RW4 vs. CW4: > 0.999 RW5 vs. RW6: 0.024 RW5 vs. CW2: 0.065 RW5 vs. CW3: 0.035 RW5 vs. CW4: > 0.999 RW6 vs. CW2: > 0.999 RW6 vs. CW3: > 0.999 RW6 vs. CW4: 0.086 CW2 vs. CW3: > 0.999 CW2 vs. CW4: 0.007 CW3 vs. CW4: 0.005	– – – – – 3.79 – 5.61 – – – – – − 5.31 − 6.72
BL (AU)	510 ± 46	512 ± 37	756 ± 112	526 ± 74	587 ± 59	601 ± 39	RW4 vs. RW5: > 0.999 RW4 vs. RW6: 0.266 RW4 vs. CW2: > 0.999 RW4 vs. CW3: > 0.999 RW4 vs. CW4: > 0.999 RW5 vs. RW6: 0.587 RW5 vs. CW2: > 0.999 RW5 vs. CW3: > 0.999 RW5 vs. CW4: > 0.999 RW6 vs. CW2: 0.064 RW6 vs. CW3: > 0.999 RW6 vs. CW4: > 0.999 CW2 vs. CW3: > 0.999 CW2 vs. CW4: > 0.999 CW3 vs. CW4: > 0.999	– – – – – – – – – – – – – – –

*RW4* regular weeks with four training sessions, *RW5* regular weeks with five training sessions, *RW6* regular weeks with six training sessions, *CW2* congested weeks with two training sessions, *CW3* congested weeks with three training sessions, *CW4* congested weeks with four training sessions, *D1* distance covered between 0 and 5.9 km/h, *D2* distance covered between 6 and 9.9 km/h, *D3* distance covered between 10 and 15.9 km/h, *D4* distance covered between 16 and 23 km/h, *D5* distance covered between > 23 km/h, *AccZ1* Accelerations Zone1 (< 2 m/s^2^), *AccZ2* Accelerations Zone2 (2 to 4 m/s^2^), *AccZ3* Accelerations Zone3 (> 4 m/h^2^), *DecZ1* Decelerations Zone1 (< − 2 m/h^2^), *DecZ2* Decelerations Zone2 (− 2 to − 4 m/h^2^), *DecZ3* Decelerations Zone3 (> − 4 m/h^2^), *BL* body load.

**Figure 1:**
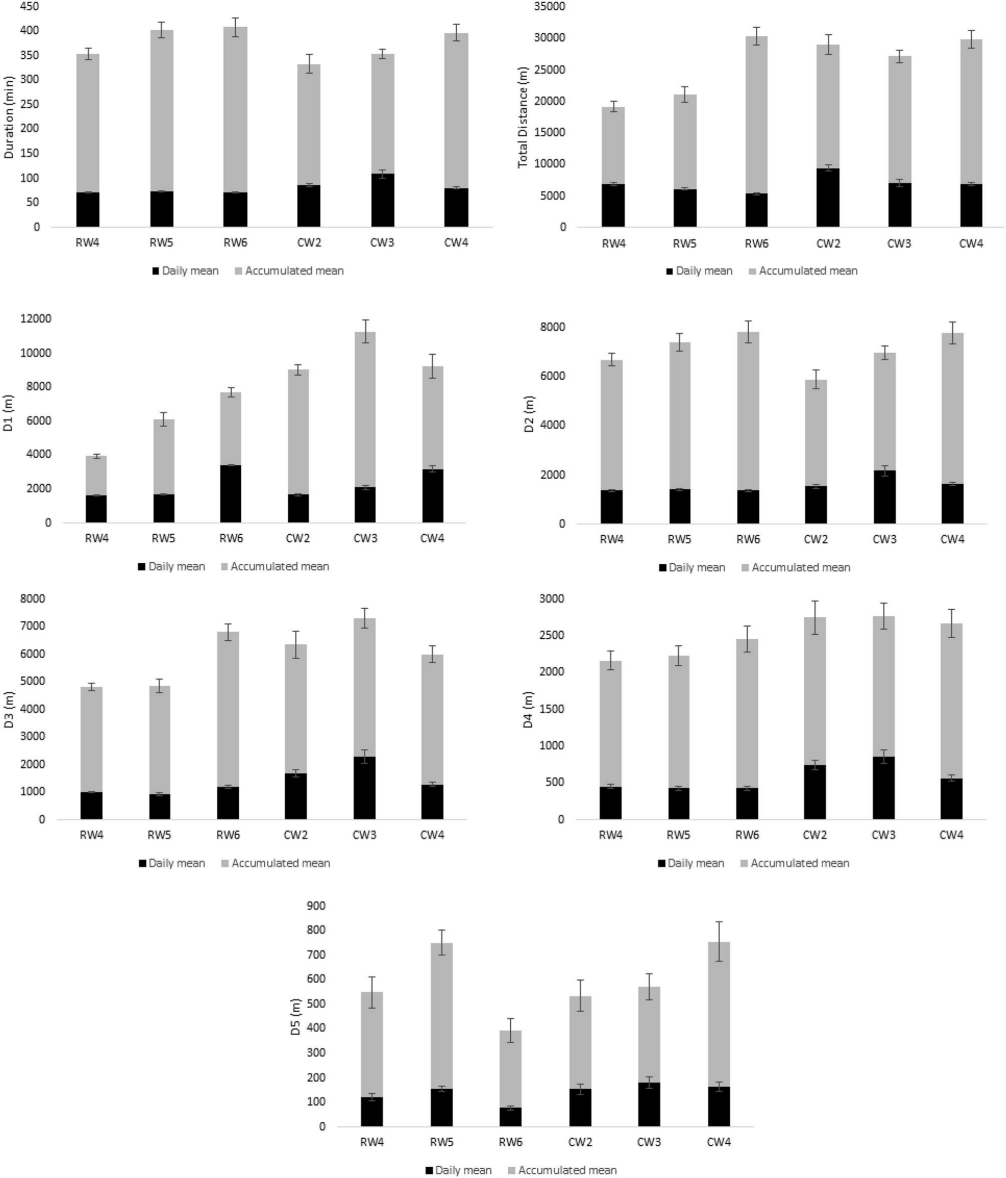
Characterization of duration and running distances measures of the six microcycles. *RW4* regular weeks with four training sessions, *RW5* regular weeks with five training sessions, *RW6* regular weeks with six training sessions, *CW2* congested weeks with two training sessions, *CW3* congested weeks with three training sessions, *CW4* congested weeks with four training sessions, *D1* distance covered between 0 and 5.9 km/h, *D2* distance covered between 6 and 9.9 km/h, *D3* distance covered between 10 and 15.9 km/h, *D4* distance covered between 16 and 23 km/h.

**Figure 2:**
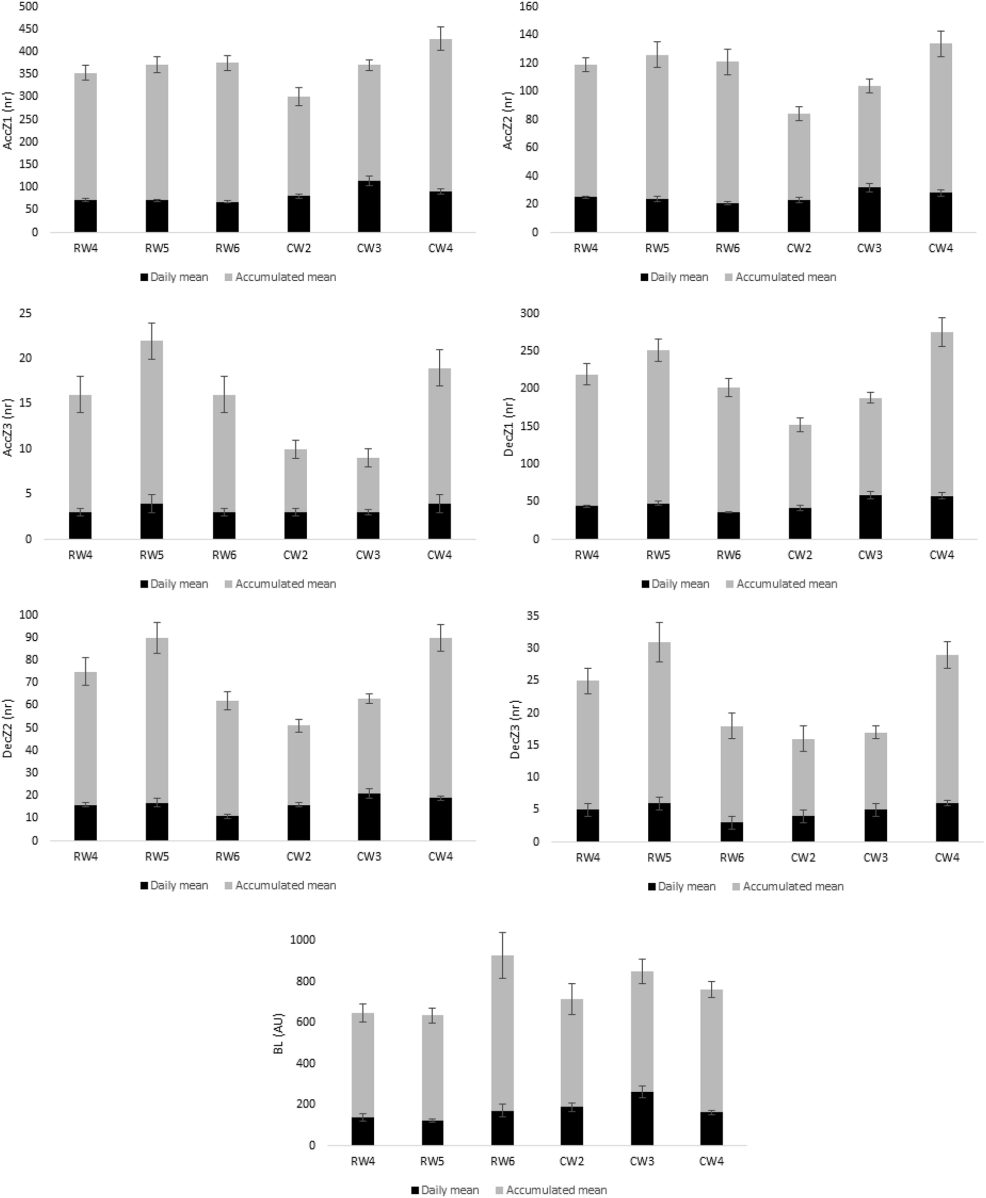
Characterization of accelerometry based measures of the six microcycles. *RW4* regular weeks with four training sessions, *RW5* regular weeks with five training sessions, *RW6* regular weeks with six training sessions, *CW2* congested weeks with two training sessions, *CW3* congested weeks with three training sessions, *CW4* congested weeks with four training sessions, *AccZ1* Accelerations Zone1 (< 2 m/s^2^), *AccZ2* Accelerations Zone2 (2 to 4 m/s^2^), *AccZ3* Accelerations Zone3 (> 4 m/h^2^), *DecZ1* Decelerations Zone1 (< − 2 m/h^2^), *DecZ2* Decelerations Zone2 (− 2 to − 4 m/h^2^), *DecZ3* Decelerations Zone3 (> − 4 m/h^2^), *BL* body load.

For quick and clear demonstration of the results, Figs. 1 and 2 show daily mean and accumulated mean values per each microcycle and per each measure.

## Discussion

This study aimed to compare distance and accelerometry-based measures in six different microcycle schedules with both RWs and CWs in professional soccer players. The researchers in this research hypothesised that in all CWs, which include more matches than RWs, higher levels of external load (such as total distance, different running speed distance, and number of accelerations/decelerations) were given to the players compared to RWs. A significant part of this hypothesis was confirmed according to the obtained results.

From the present results, for total distance and duration data in the weekly mean, CWs reported higher values in all sections than RWs. Given that there were two matches in the CWs compared to the RWs (only one match), these results are justifiable because the players in this study were the starter players. The matches always started with these players, who have been on the field longer than non-starter players. It seems that they spent more time training and competing during the week. On the other hand, each player ran between 10 and 13 km in each match. Therefore, considering that they have played more matches in CW microcycles, they have convered more distance.

The results also showed in distance and duration parameters that CW4 behaves differently than CW2 and CW3. In the duration data, RW5 and RW6 were significantly higher on accumulated mean values than CW3. Because CW3 had higher weekly mean values than RW5 and RW6, it had lower accumulated mean values. Considering that in CW3, the players have participated in two matches and the amount of running in the matches is usually high, it seems that the reduction of the accumulated average in this microcycle is due to the decrease in the training load in the training sessions after the competitions due to the better recovery of the players and the players spent less time training in these sessions and also performed exercises with less load. It is also possible that due to the compression of the matches, non-starter players may be used instead of starter players in the CW3 microcycle, which will reduce the playing time of the starter players. It should be emphasized that only data about the team's primary players were used in this analysis. In the total distance data, on accumulated mean values, RW4 had lower values than all other macrocycles. RW4 included 11 weeks in which 11 matches and 44 training sessions were performed, in which one match and four training sessions per week were performed. As a result, it had fewer matches than the CWs, and given that most running distances occur in matches, RW4 reported more periodic distance than the CWs. On the other hand, among RWs, RW4 had fewer sessions per week and as a result, reported less distance than the rest.

Howle et al.^31^ who looked at the injury associated with increased training load in single match and multi-match weeks over three seasons in soccer, reported that total distance and total duration decreased during congestive weeks. These findings are in direct opposition to those of the current investigation. The following reasons for the disagreement between Howle et al.^31^ conclusions and the results of the present study are: in the Howle et al.^31^ study, 42 players were evaluated over three seasons, and according to the researchers, they did not have part of the GPS data throughout those three seasons while in the present study, only the team’s main players were surveyed during a season and all GPS data was available. Since bench and non-squad players reported almost no information during the match, integrating their data with the data of the key players can reduce the average total external load in the previous research^31^.

The study of Clemente et al.^32^ compared distance-based load indices during congested fixture periods among different levels of participation in matches. The results of this study showed that the total distance traveled in the weeks when two matches were held in more than the weeks when one match was held, and also in the weeks when three matches were held, the total distance traveled was more than the weeks when two matches or only one match has been held. These findings were in line with the results of the present study.

In the data related to D1, D2, and D3, weekly mean and accumulated mean values, CWs reported higher values than RWs. Considering that in CW microcycles, more matches were played per week than in RWs, and in each match, soccer players performed the highest number of sprints at different speeds to catch the ball or dribble, or in running courses, consequently, in CW microcycles, the amount of running at different speeds is higher than in RWs, and the results obtained from this study can be justified^33^. These findings can justify that players had covered long distances at high speed. The only exception is RW6, which offered higher values than CW2 and CW3 in D1 and D2. During this microcycle, four weeks, 24 training sessions and four matches were held, which averaged one matches and there were six training sessions per week in this microcycle. This training, match density, and probably coaching approaches have increased the running speed and distances traveled at high speeds in the two sections D1 and D2, in the RW6.

In D4 (16–23 km/h) for the weekly mean data, all CWs were significantly higher than all RWs, and such as D1, D2, and D3, what can be justified due to the large number of matches in CW. In D4 accumulated mean values, RW6 was significantly higher than RW5 and RW4. According to what is mentioned in the sections above, RW6 had an average of more training sessions and matches per week than other RWs, and consequently, players who had played more, also experienced more sprints and more distance traveled at high speeds than other RW microcycles.

D5 (distance covered > 23 km/h) in both weekly mean and accumulated mean values data showed differences between different microcycles but it cannot be claimed that CWs presented higher values than RWs or vice versa. Running distances above 23 km/h and sprints at higher speeds in training and competitions are rare^33^. For this reason, the difference between the microcycles in D5 can depend more on the difference in the exercises performed during the microcycles or the importance of the matches performed (official league matches, knockout matches, or friendly matches) in the microcycle. The findings of Clemente et al.^32^ were consistent with the present results. Clemente et al.^32^ found that high-speed running during weeks with two matches is much greater than during weeks with only one match (79%). In this sector, the weeks with three matches differed significantly from those with one match (60%)^32^.

Penas et al.^34^ found that those who played two matches a week covered shorter distances at maximal (> 23 km/h), submaximal (19.1–23 km/h), and medium (14.1–19 km/h) intensities than those who played one match during the week, but the difference between the two groups was not significant. These findings were not consistent with the present study. One of the reasons for the inconsistency of results is that in the study of Penas et al.^34^, 27 players from the same team participated, which, unlike the current study, also includes bench players, and as mentioned earlier, the data of these players can have a detrimental effect on the overall data. Penas et al.^34^ also suggested that insufficient recovery and accumulated fatigue may have led to such results. The researchers also said that running at high speeds depends on the competition level. For instance, in matches where elite players ran at speeds less than 19 km/h, the team lost and reported that the host teams ran shorter distances at maximum speed than the visiting teams^34^. The findings of this study were in line with the results of the present study in terms of distances traveled at speeds above 23 km/h, even though the present study did not address match results/location. It seems that the workload applied in the training as well as the tactical needs and the importance of the competition have an effect on the players' efforts to run at the highest speed in long distances, and the more workload the coach's plans have in training and the need for sprints in long distances. The longer it is, the more influential the match is to the players, the more effort they will make, the more sprints they will run, and the more distances they will run at a high speed to achieve the best results in matches and training.

In the accelerations and decelerations section of the weekly mean data in Zone 1, all CWs showed higher values than the RWs, but this difference was statistically significant at CW3 and CW4. Due to more matches in CW microcycles and the need for acceleration and deceleration in different positions of the match for dribbling and ball possession and competition to create the position^35,36^, players presented higher values of accelerations and decelerations. But these differences were less in AccZ2, and in the acceleration section, only CW4 was significantly higher than RW6, while in the deceleration section, CW4 was considerably higher than RW4. Also, CW3 and 4 were larger than RW6.

In AccZ3, these differences were eliminated, to the extent that RW5 was larger than CW2, and in Zone 3 deceleration, CW3 and CW4 were still larger than RW6. According to the present study's findings, the presence of many matches per week has the greatest impact on the acceleration and deceleration of zone one, and this difference is less in other zones.

Despite all findings, this study has some limitations. First, the results come from a case study with a small number of players from Iran. Thus, generalization is limited, and more research should be performed to confirm the present analysis. Internal load measures were also not used, which could provide better insights and should be recommended in future studies. Also, it would be relevant to analyse if ratios between training and matches differ, considering the different training schedules. Finally, other studies can use the present example and test if the higher loads from the CWs contribute to injuries. Such analysis would provide relevant information to training periodization. For instance, higher loads can be applied in RWs if no injuries were found.

## Conclusions

The results showed that the exact measurements (e.g. high-speed running between 16 and 23 km/h and acceleration > 2 m/s^2^) can lead to different results when analyzed using daily mean or weekly accumulated mean values. This suggests that both types of analysis should be performed in future studies. Nonetheless, our research showed a trend towards higher CW values than RW, providing information on organising the load in different weekly scenarios.

Given that the external stress on CW increases significantly in players who participate in more matches, coaches should pay special attention to players who participate in matches to reduce fatigue and improve their performance. Ensuring recovery mechanisms are in place to minimize the higher external load is recommended. Additionally, it can be suggested that coaches equialize training sessions for non-starters who experience lower external load than starter players. Also, coaches and/or strength and conditioning professionals should keep the external load of all players balanced and avoid the tightness of the matches during the weeks of the season to maintain their health, reduce fatigue, and prevent non-functional overreaching syndrome or non-contact injuries.

## Data Availability

The datasets generated during and analyzed during the current study are available from the corresponding author on reasonable request.
